# MicroRNAs and their role in the malignant transformation of oral leukoplakia: a scoping review

**DOI:** 10.4317/medoral.24975

**Published:** 2021-09-25

**Authors:** Sven Niklander, Daniela Guerra, Felipe Contreras, Wilfredo González-Arriagada, Constanza Marín

**Affiliations:** 1 DDS, MDent, MSc, PhD. Associate Professor. Departamento de Patología y Medicina Oral, Universidad Andres Bello, Viña del Mar, Chile; 2 DDS. Instructor. Departamento de Patología y Medicina Oral, Universidad Andres Bello, Viña del Mar, Chile; 3 DDS, MSc, PhD. Associate Professor. Facultad de Odontología, Patología Oral y Maxilofacial, Universidad de los Andes, Santiago, Chile; 4 DDS, Msc. Assistant Professor. Departamento de Patología y Medicina Oral, Universidad Andres Bello, Viña del Mar, Chile

## Abstract

**Background:**

MiRNAs are small non-coding RNAs that regulate gene expression at the post-transcriptional level and have been associated with malignant transformation of oral epithelial precursor lesions such as oral leukoplakia. The aim was to perform a scoping review of the contemporary literature about the different roles of miRNAs during the malignant transformation of oral leukoplakia.

**Material and Methods:**

We conducted a systematic search with the following MeSH terms: ‘oral leukoplakia’, ‘carcinoma in situ’, ‘microRNAs’, ‘mouth neoplasms’ and ‘epithelial–mesenchymal transition’ in PubMed/MEDLINE, EMBASE and SpringerLink.

**Results:**

Fifteen articles were included for analysis, among which *in vivo* and *in vitro* articles were included. A total of 21 different miRNAs were found to be involved in the malignant transformation process of oral leukoplakia. Regarding their possible effects, 6 miRNAs were classified as oncogenic, 5 as tumour suppressors and 10 were related to epithelial–mesenchymal transition, invasion and migration.

**Conclusions:**

Based on the current review, we concluded that miRNAs-21, 345, 181-b and 31* seem to be potential markers of malignant transformation of oral leukoplakia. However, further clinical prospective studies are needed in order to validate their utility as prognostic biomarkers.

** Key words:**miRNAs, oral leukoplakia, oral squamous cell carcinoma, biomarkers, malignant transformation.

## Introduction

Oral squamous cell carcinoma (OSCC) is the most common malignancy of the head and neck (HN) (1). Due to late diagnosis, OSCC is associated with significant morbidity and mortality, with a 5-year survival of less than 50% (2,3). Most OSCC cases are preceded by precursor lesions, collectively referred to as oral potentially malignant disorders (OPMDs) (2-4). The OPMD group comprises many disorders with an increased risk of malignant transformation (MT) and includes erythroplakia, leukoplakia, submucous fibrosis and oral lichen planus, among others. Oral leukoplakia (OL) is the most common OPMD (4,5) and its prevalence depends on the studied population, but usually ranges from 0.4 to 2.6%, with a MT rate of 3–17.5% (3,5). Currently, the risk of MT of an OL is estimated by the degree of dysplasia and the clinical form (homogeneous, non-homogeneous and proliferative verrucous leukoplakia), although other clinical and histopathological parameters can be useful (4-7). The majority of the molecular markers associated with the MT of OPMD are addressed to the classical hallmarks of cancer (8), however, none of these are sufficient to predict the MT of OL. Therefore, histopathological analysis is still being used as the predictive gold standard (4,7). MiRNAs are single-stranded non-coding RNA molecules (of approximately 22 nucleotides in length) that target mRNA molecules and suppressing their expression, by either translational modifications or mRNA cleavage (9). Due to its potential impact in the oral carcinogenesis process, we aimed to perform a review of the different roles of miRNA during the MT of OL.

## Material and Methods

This scoping review was performed according to the Preferred Reporting Items for Systematic Reviews and Meta-Analysis (PRISMA) guidelines and scoping review guidelines from the Joanna Briggs Institute (10) . We summarize the utility of microRNAs in predicting the malignant transformation of oral leukoplakia basing our search on the following MesH terms: ("MicroRNAs"[Mesh]) AND "Leukoplakia, Oral"[Mesh]; and ((("Leukoplakia, Oral"[Mesh]) OR "Carcinoma in Situ"[Mesh]) NOT "Uterine Cervical Neoplasms"[Mesh]) AND "MicroRNAs"[Mesh]. In addition, another search strategy was conducted with the following keywords: "epithelial mesenchymal transition" AND "microRNAs" AND "oral cancer"; "epithelial mesenchymal transition" AND "microRNAs" AND "oral squamous cell carcinoma"; "oral leukoplakia" AND "microRNAs" AND "oral squamous cell carcinoma"; "oral leukoplakia" AND "microRNAs" AND "malignant transformation"; "oral dysplasia" AND "microRNAs" AND "progression”; "oral leukoplakia" AND "microRNAs" AND "progression"; "microRNAs" AND "progression" AND "oral squamous cell carcinoma".

- Inclusion criteria

Selected articles were considered eligible when they fulfilled the following criteria: a) clinical studies whose content was associated with miRNAs as predictive markers of the MT of OL and/or oral dysplasia with follow-up; b) *in vitro* studies who assessed epithelial-mesenchymal transition; c) articles in English; d) articles that were published in a 10-year period before this study was conducted (April 2009–April 2019); e) articles with positive cases corroborated by histopathological analysis.

- Exclusion criteria

Exclusion criteria were the following: a) articles describing miRNA extracted exclusively from blood, plasma, saliva, or serum; b) review articles, book chapters, systematic reviews and meta-analyses; c) articles published before or after the established dates; d) studies based only on bioinformatics predictive approaches without experimental analysis.

Selection of the articles was made using the following databases: PubMed/MEDLINE, EMBASE and SpringerLink. This was further supplemented through hand search and by screening the entire reference lists of included studies. All the extracted data were registered in a specifically designed spreadsheet to eliminate possible mistakes.

## Results

**Figure 1:**
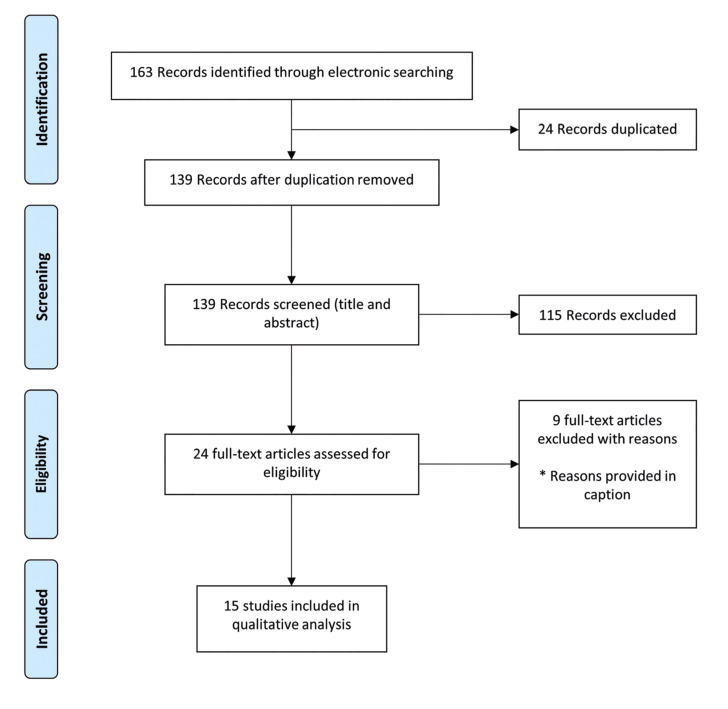
PRISMA flow diagram. Nine articles were excluded with the following reasons: no follow-up (n=4), reviews (n=3) and bioinformatic analysis only (n=2).

Initially, 163 studies were identified in the literature search. Following duplicate removal (n=24), 139 studies had their titles and abstract screened by two reviewers (FC and DG) to ensure they satisfied the inclusion and exclusion criteria. If there was any doubt at this stage, the studies were included for full-text review. One hundred and fifteen articles were excluded as they were not relevant, and 24 articles had a full-text review. Any disagreements regarding inclusion/exclusion were resolved through discussion and the input of a third reviewer (CM). Nine articles were excluded with the following reasons: no follow-up (n=4), reviews (n=3) and bioinformatic analysis only (n=2). Finally, 15 articles met the inclusion criteria and were included in this scoping review (Fig. 1).

From the 15 selected articles, 4 were studies performed exclusively using patient tissue samples (including OL with/without dysplasia, carcinoma in situ, OSCC and normal oral mucosa) corresponding either to retrospective or prospective studies. These articles assessed oral cancer progression by comparing miRNA expression between progressive and non-progressive epithelial lesions, with follow-ups that ranged from 1 to 5 years (11-14). Five articles were a combination of *in vitro*/in vivo studies (15-19) and the other 6 were exclusively *in vitro* (20-25) (Supplement 1).

**Figure 2:**
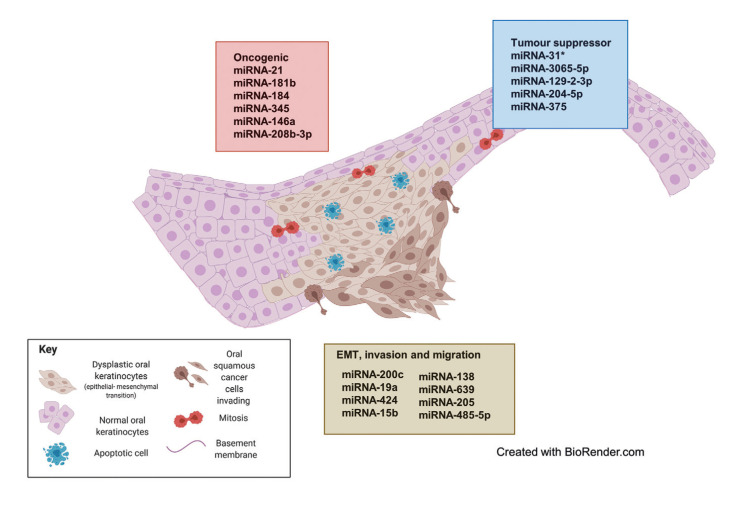
MiRNAs involved in malignant transformation of oral leukoplakia. The oncogenic (red box- red cells represent mitosis and proliferation) and tumour suppressor (blue box- blue cells represent apoptosis) miRNAs are based on the results of studies performed on progressive OLs. On the other hand, miRNAs associated to EMT, invasion and migration (brown box- brown cells represent EMT and invasion) are extracted from experimental studies on cell lines. Details about their regulations and targets are on Table 1.

Of the selected studies, 6 performed a genome-wide miRNA expression approach to detect candidate miRNAs that were confirmed by qRT-PCR analysis (11,14,17-20). The remaining articles determined the expression of specific miRNAs based on previous results and/or bioinformatic tools. Most of the *in vivo* studies aimed to compare miRNA expression levels between progressive and non-progressive OL without further analysis of possible miRNA targets (11-14,19,26). In vitro studies showed a much deeper analysis of miRNA expression and possible functions, including migration, invasion and proliferation assays after miRNA overexpression or knockdown (Supplement 1). A total 21 different miRNAs were analysed in terms of their expression levels, possible effects in (early) oral carcinogenesis and their potential targets (genes or pathways; Table 1). Regarding their possible effects, 6 miRNAs were classified as oncogenic, 5 as tumour suppressors and 10 were related to epithelial–mesenchymal transition (EMT), invasion and migration (Fig. 2).

**Table 1:**
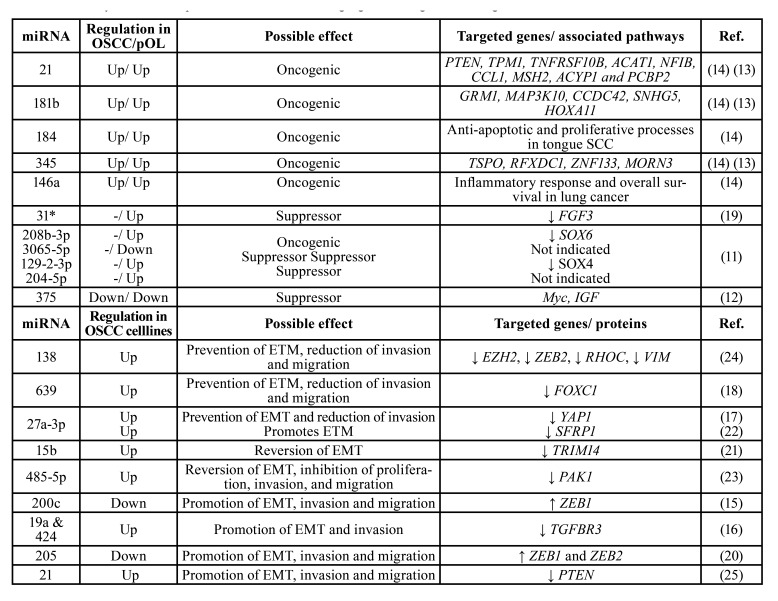
Summary of miRNA expression, their effects and target genes during oral carcinogenesis.

- Oncogenic-related miRNAs

In this article, we will term as oncogenic those miRNAs that are specifically believed to target genes that stimulate cell proliferation and/or inhibit apoptosis (14). Overall, three articles reported overexpression of miRNAs considered to be oncogenic (11,13,14). As expected, oncogenic miRNA expression increased during the MT of OLs (with/without dysplasia) towards OSCC (Table 1). MiRNAs-21, 181b, 184, 345 and 146a were found to be overexpressed in progressive OL and OSCC. However, in a linear regression analysis, miRNAs-21, 181b and 345 showed to be upregulated gradually from progressive OL to OSCC, but not in non-progressive OL; thus, they were proposed as tentative biomarkers for the early MT of OL (14). One study identified upregulation of miRNA-21 in OL and OSCC compared to normal oral mucosa and tried to associate miRNA-21 expression with the severity of epithelial dysplasia, but no significant association was found (13). In another article, upregulation of miRNA-208b-3p was shown in the progressive group of OL compared to the non-progressive group (11). This miRNA has a presumptive oncogenic role since is associated with the downregulation of SOX-6 protein, which is considered a tumour suppressor protein (11).

- Tumour suppressor-related miRNAs

Three articles reported the deregulation of tumour suppressor-related miRNAs (11,12,19) such as miRNAs-31*, 3065-5p, 129-2-3p, 204-5p and 375 (Fig. 2). MiRNA-375 was downregulated in OSCC and progressive OLs compared to non-progressive lesions, suggesting its role as a tumour suppressor during oral carcinogenesis, probably through the regulation of the expression of Myc and insulin-like growth factor (IGF) oncogenes (12). Inversely-related tendencies were seen between the upregulation of miRNAs-31* and 129-2-3p; and the downregulation of their targets FGF3 and SOX4 respectively (Table 1) (11,19).

- Epithelial–mesenchymal transition, invasion, and migration-related miRNAs

Ten studies identified miRNAs related to EMT, invasion and/or migration. Of these, 6 were performed only *in vitro* with OSCC cell lines and in most cases, using human normal keratinocytes as controls (20-25), whereas four worked with cell lines and OSCC/OL tissue samples (Supplement 1) (15-18). EMT induction was usually assessed by measuring the expression of EMT-related proteins such as E-cadherin, vimentin and N-cadherin. In this category, miRNAs-138; 639; 27a-3p; 15b; 485-5p; 19a and 424; and 21 were found to be upregulated, whereas the expression of their corresponding target genes EZH2, ZEB2, RHOC, VIM; FOXC1; YAP1, SFRP1; TRIM14; PAK1; TGFBR3; and PTEN respectively, were downregulated (Table 1) (16-18,21-25). On the contrary, miRNAs-200c and 205 appeared as downregulated in OSCC cell lines and tumour samples (the former) and in OSCC cell lines (the latter) (15,20), whereas their targets, ZEB1 (for miRNA-200c) and ZEB1 and ZEB2 (for miRNA-205), showed to be upregulated. As shown by both research groups, the overexpression of miRNA-200c and miRNA-205 was associated with the inhibition of EMT, cell migration and invasion in oral cancer cell lines (15,20). In general terms, overexpression of these miRNAs was related to the prevention of EMT and the reduction of cell invasion and cell migration. Nevertheless, upregulation of some of these miRNAs has also been associated with the contrary effect. This is the case of miRNA-21 which showed to be upregulated in OSCC cell lines, promoting EMT along with proliferation and migration through the downregulation of its target PTEN (25). MiRNAs-27a-3p is controversial, as opposite functions have been reported. One study found miRNA-27a-3p to prevent EMT and to reduce cell invasion by downregulating YAP1 (17), whereas another study found that this miRNA promotes EMT by downregulating SFRP1 (22). Another article showed that miRNAs-19a and 424 promote EMT and the invasion of Cal-27 cells (human tongue squamous cell carcinoma cell line) by downregulating TGFB3 (16). On the other hand, miRNA-15b was shown to downregulate TRIM14 expression, which leads to the promotion of MET (mesenchymal–epithelial transition) and the reversal of cisplatin chemoresistance of cisplatin-resistant SCC25 cells (21). Similar results were found in the interaction between miRNA-485-5p and its target PAK1 (23); thus, the upregulation or the restitution of these miRNAs in OSCC cell lines reversed the EMT and inhibited invasion and migration (21,23).

- MiRNAs as markers of malignant transformation

Two articles analysed the predictive value of specific miRNAs as possible markers for the risk estimation of MT in dysplastic lesions (11,12). These studies generated ROC curves for miRNAs-375 (12), 208b-3p, 204-5p, 129-2-3p and 3065-5p (11). One study concluded that the expression level of miRNA-375 has prognostic potential in detecting MT and in distinguishing between progressive and non-progressive premalignant lesions (12). The other study suggested a miRNA marker panel (which included miRNAs-208b-3p, 204-5p, 2-3p and 3065-5p) to be used in combination with other variables, such as age and histopathological diagnosis (11). The authors reported a strong predictive value for MT when combining these three variables, with a sensitivity of 76.9% and a specificity of 73.7%, which performed better than histology by itself (11).

## Discussion

The development of genetic and molecular biology techniques has revolutionised the diagnosis and characterisation of oral cancer and its precursor lesions. Nevertheless, there are still no molecular markers that are able to predict which OPMDs are more likely to transform into OSCC and can be used in clinical settings (4). During recent years, epigenetics has generated special attention among researchers and miRNAs have been proposed as potential biomarkers for MT of OPMDs, as in most cases oncogenic miRNA expression in OL and OSCC is high (27). In the present scoping review, we classified all miRNAs into three groups, based on the events that occur during the MT of an OL: 1) oncogenic-related miRNAs; 2) tumour suppressor-related miRNAs; 3) EMT-, invasion- and migration-related miRNAs. Oncogenic miRNAs are commonly upregulated during OSCC development and have been related to the acquisition of a malignant phenotype. MicroRNA-21 is a good example of an oncogenic miRNA as it showed to be overexpressed in progressive OL and OSCC (13,14,19). MiRNA-21 is closely related to the proliferation and inhibition of apoptosis (13,14,19). Different reports have suggested that this miRNA would participate in invasion and metastasis by acting on tumour suppressor genes closely related to DNA repair, such as: tropomyosin I (TPM1), serpine peptidase inhibitor clade B (ovalbumin), Serpin B family member 5 (SERPINB5) (14,19) and phosphatase and tensin homolog gene (PTEN) (13,25). Zeng *et al*., reported that a long non-coding RNA (lncRNA) named Growth-arrested-specific transcript 5 (GAS5), showed to negatively regulate miRNA-21, promoting PTEN expression which subsequently inhibit the PI3K/Akt pathway (25). The latter corresponds to an important pathway that regulates cell cycle. The authors concluded that GAS5 may be a novel therapeutic target for OSCC. Similarly, Chattopadhyay *et al*., reported that miRNA-31 participates in all early stages of oral carcinogenesis, increasing cell proliferation, inhibiting apoptosis, promoting EMT and invasion (28). Using bioinformatics, they estimated that miRNA-31 targets DMD, CXCL12 and WASF3. It has been proposed that miRNA-31 could be related to cell immortalisation and disruption of DNA repair genes, favouring genomic instability and EMT (28).

In relation to tumour suppressor miRNAs, the findings are more variable as their expression is more dependent on the nature of the lesion. A striking result in regard to miRNA-31* was reported (19), because it was found to be upregulated in both progressive OL and OSCC; however, the authors classified it as a tumour suppressor-associated gene in oral carcinogenesis. This statement was based on the functional analysis of three cell lines with different malignant potential; therefore, the function of this miRNA should be carefully addressed. They observed the effects of miRNA-31* in two precancerous cell lines (Leuk-1 and HIOEC) and one cancerous cell line (Cal-27), from which they concluded that this miRNA enhanced apoptosis and decreased migration and invasion abilities (in the three cell lines), inhibited cell proliferation in HIOEC and increased the percentage of Cal-27 cells in the S/G2 phase of the cell cycle after the transfection with miRNA 31* inhibitor (19). Another unexpected result was seen with the expression levels of miRNAs-129-2-3p and 204-5p. Both miRNAs were shown to be upregulated in progressive OL, validated by qRT-PCR (11); however, they were shown to be downregulated when assessed by deep sequencing. Because their putative role as tumour suppressor is recognised, the authors classified them as such (11), and so did we. MiRNA-129-2-3p targets and negatively regulates the expression of SOX4, an oncogene that has been associated with the progression of prostate cancer (29) and oral lichen planus to OSCC (30). Nevertheless, further analysis is needed to clarify the true nature of these miRNAs in the MT of OPMDs, especially in OL.

MiRNAs related to EMT can have opposite functions: they can prevent or promote it. MiRNAs-138 and 639 have been reported to prevent EMT and reduce cell invasion by silencing EZH2, ZEB2 and RHOC (miRNA-138) (24) and FOXC1 (miRNA-639) (18). These regulators are involved in inhibiting, directly or indirectly, cell binding proteins such as cadherins (15,18,24). In OL and OSCC, these miRNAs are under-expressed, which leads to a higher expression of the aforementioned targets and resulting in EMT (15,18,24). Downregulation of miRNA-138 induces changes in cell migration, invasion and motility, polarity loss and acquisition of a mesenchymal phenotype (24). MiRNA-138 downregulates the expression of ZEB2 and EZH2 whose are powerful EMT inductors and suppressors of E-cadherin expression. MiRNA-200c is a good example that miRNAs can have tissue-specific functions. In bladder cancer, this miRNA has oncogenic functions (31), whereas in the oral cavity it has been reported to prevent OSCC development, when overexpressed, through the suppression of ZEB1 (15), whose overexpression is associated with E-cadherin loss, related to enhancement of invasion and migration. In the same way, miRNA-127a-3p has shown different effects depending on its targets. In the current revision, it showed to prevent EMT when acting on the YAP1-OCT4-Sox2 signalling axis or to promote it when interfering with the SFRP1-via Wnt/β-catenin signalling pathway (17,22). MiRNAs are small non-protein coding RNAs that can regulate gene expression (32-35). However, one miRNA can regulate more than one gene, and that gene may be targeted by more than one miRNA (20,24,26,35,36). The fact that the same miRNA can have different effects on different tissues and/or different stages of the same pathology is a good example of the complex scenario of miRNAs in cancer biology (14,28,31). Currently, miRNAs are being assessed for their potential as molecular therapeutic targets in oral cancer since the overexpression and downregulation of certain miRNAs have been associated with chemoresistance. As mentioned before, Wang *et al*. reported that the upregulation of miRNA-15b inhibited the formation of a cancer-initiating cells (CIC), promoted MET and sensitised oral cancer cells resistant to cisplatin (SCC25-res cells) by targeting TRIM14 (21). Correspondingly, miRNA-485p overexpression inhibited PAK1 protein expression in SCC25 cells, reverting cisplatin-resistance in SCC25 cells (23). This is a promising and an ongoing approach that needs to be further elucidated in longitudinal studies, with larger cohorts in oral cancer and OL (or OPMDs) patients. As it was shown, miRNAs mediate the activation or suppression of important signalling pathways in the pathogenesis of oral cancer and this review evidence that the study of the role of miRNAs in the MT of OL needs further development. Based on the current review, we concluded that miRNAs-21, 345, 181-b and *31 seem to be potential markers of MT of oral leukoplakia, as supported by their reported role in clinical studies that worked with progressive and non-progressive OL, and further supported by their role in the carcinogenesis in other cancers. Furthermore, as Philipone *et al*. proposed, a panel of microRNAs could be used as possible biomarker, along with other variables such as age and histopathological diagnosis, in order to determine the predictive value for MT in OL (11). This statement is further supported by El-Sakka *et al*, who concluded that the prognostic utility of microRNAs in predicting malignant transformation of OPMD is equivocal and a combined set of microRNAs should be considered (37). Although all miRNAs involved in EMT underwent strict experimental analysis, all these studies were made *in vitro*; thus, the results need to be confirmed by clinical research. Other reviews have reported the role of miRNAs as biomarkers in the progression of potentially malignant disorders, focusing on the use of microRNAs in body fluids; or have been focused on the bioinformatic analysis of primary articles showing the gene ontology and/or signalling pathways associated to the putative targets of the miRNAs. An ongoing field at the moment is the assessment of oral swirls, which allows the great advantage of using a non-invasive and rapid collection method of salivary miRNAs. Results have shown high-accuracy in the detection of cancer-related miRNAs in early stages of oral carcinogenesis such as the upregulation of miRNA-21 and 31 and the downregulation of miRNA-99a, 125b and 100 (38,39), however longitudinal studies with larger cohorts are needed to validate these results. Our article aimed to review the current literature for studies which assessed the utility of microRNAs (expressed by tissues) in the prediction of the malignant transformation of oral leukoplakia, complemented with *in vitro* articles whose findings specifically addressed EMT. We realised that there is a lack of clinical articles with follow-up of OL and/or oral dysplasia. This is further supported with the findings of El-Sakka *et al* who reported that from a total of 40 articles included in their systematic review, only 11 considered biomarkers for OL. The authors highlighted the variability among the studies due to different follow-up periods (or no follow-up), unpaired samples, use of controls, among others (37). In the same way, as reported by Villa *et al*, there are major limitations among the studies about prognostic biomarkers in OL, preventing their clinical applications, including small sample sizes, lack of histopathological and demographics data, limited and variable follow-up information and/or non-inclusion of a control group (40). Finally, we think that normal oral mucosa and hyperplastic epithelium should be characterised in terms of their miRNA expression and compared with OSCC and progressive and non-progressive OL to detect early deregulations of miRNAs before the development of the clinicopathological alterations.
